# Bronchiectasis

**Published:** 1885-10

**Authors:** John O. Roe

**Affiliations:** Rochester, N. Y.; Fellow of the American Laryngological Association, Member of the Medical Society of the State of New York, of the American Medical Association, etc., etc.


THE BUFFALO Medical AND Surgical Journal

VOL. XXVI. OCTOBER, 1885. NO. 3.

### Original Communications.

### BRONCHIECTASIS.

A CASE IN AN ADULT, THE RESULT OF CHRONIC BRONCHITIS CONTRACTED IN INFANCY: FOURTEEN OUNCES OF CLEAR PUS EXPECTORATED DAILY: PATIENT CURED BY THE PERSISTENT EMPLOYMENT OF ATOMIZED INHALATIONS AND ARTIFICIALLY RAREFIED AIR.*

BY JOHN O. ROE, M. D., Rochester, N. Y.

Fellow of the American Laryngological Association, Member of the Medical Society of the State of New York, of the American Medical Association, etc., etc.

H., twenty-five years of age, the editor of a county paper, came under my observation September 26, 1879.

He was of light complexion, of sanguine temperament, 5 ft. 11 in. in height, of slender form, thin in flesh, and weighed 123 lbs. From childhood, he had been what is termed delicate.

When two years old, as a result of frequent colds to which he was subject, he began to have a bronchial trouble, attended by cough and expectoration.

* Read before the Medical Society of the State of New York, February 5, 1885.

When six years old, he had scarlet fever during the early autumn; when eight years old, he had inflammation of the lungs. The following winter was spent in Brooklyn. The next spring he was in a very much debilitated condition, when Dr. Willard Parker was consulted in regard to him.

During that summer, he had night sweats and hectic, from which he slowly recovered, but his health was poor and uncertain most of the time. When he was sixteen, he spent a summer engineering, which greatly invigorated him, and his health remained fairly good, until he was twenty-four, although he was subject to frequent attacks of congestion of the lungs, and was never free from bronchial cough and expectoration. During December, 1878, he had typhoid fever mildly, without increasing the expectoration, but it left his health in an impaired condition.

During April and May, 1879, he had five different appearances of purpura hæmorrhagica. He took a sea voyage to Charleston, S. C., and remained a month on the coast and saw no more of the purpura.

The 1st of the following June, he had an acute laryngitis, the result of a cold, which was followed by frequent, violent and long-continued paroxysms of coughing, that greatly increased the expectoration (from 5 to 14 oz. daily).

About the 15th of August, he observed that his expectoration was of a red brick-dust color for the greater portion of each day. This continued at the time I saw him, six weeks later.

Physical examination: By the laryngoscope, showed chronic catarrhal laryngitis and tracheal catarrh. The trachea was very straight, and pus could be seen at the bifurcation. After clearing the pus out by coughing, more could be seen pouring out of the left bronchus.

By percussion, the chest was full and resonant, except a limited area of dullness just below the apex of the left lung, and this was greatly diminished after free expectoration.

By auscultation, in the left lung mucous rales were heard throughout the upper and middle lobes during quiet breathing. On forced expiration, low pitched bellows-like rales or rattles were heard under the third rib, but so loud as to be distinctly heard at any part of the chest.

These sonorous rales were evidently due to pus set vibrating by the column of air; for, when the pus had been cleared away by forced expectoration, this sound disappeared and could be heard during forcible respiration, only when there was an accumulation of pus.

During quiet breathing the respiratory sound was markedly tubular, but, after clearing away the pus, it became amphoric and cavernous. Well-marked bronchophony was also distinctly heard over this region.

In the right lung, and also in the peripheral portions of the left lung, there were emphysematous dilatations of many of the air cells with narrowing of the smaller tubes; also, some catarrhal exudation in the larger tubes.

In the upper portion of the left lung, there was a cavity, but there was no evidence that it was tubercular, as there was entire absence of any rise in temperature or other systemic disturbance. By a microscopic examination, the expectorated material was found to be composed almost entirely of pure laudable pus; the residue was blood with a little mucus. The discoloration was due to the blood which was thoroughly incorporated or mixed with the pus. This expectorated material never at any time had the slightest odor or evidence of decomposition.

Diagnosis: Bronchiectasis, a large saccular dilatation of a bronchus in the left lung about under the third rib, lined by a pyogenic membrane, and associated with a general bronchitis and emphysema.

Various opinions, the patient told me, had been given in his case.

It had been generally considered that he had simply chronic bronchitis. One physician thought the pus was due to ulceration of the trachea; while by one of our most eminent physicians it was thought there was a chronic abscess in the apex of the left lung from which the pus was discharged.

I saw no more of the patient until February 20, 1880, when he presented himself for another examination.

I found at this time some bulging and increased resonance over the apex of the left lung where there was dullness before. The same loud sonorous sound was heard on forced expiration, and evidences of increase of the cavity, and of the emphysema in the surrounding lung were apparent. Numerous sibilant rales in the smaller tubes indicated narrowing of the tubes.

As my diagnosis had not been corroborated, by my advice he went to New York City, and consulted Dr. Alonzo Clark, who, independently, made the same diagnosis that I had made.

On the same day, another distinguished physician in New York City diagnosticated the case to be one of tubercular phthisis, and, three months later, a physician in San Francisco made the same diagnosis.

In regard to treatment, all advised that he should go to California.

I told him by all means to follow the preponderance of advice, although I could not quite see how climatic influences could be expected to reduce that large bronchietic cavity, however salutary they might be for the bronchitis.

He went to California by sea, via the isthmus. The sea voyage appeared to improve his general condition. He spent most of his time, while there, in San Diego county—about six weeks on a sheep ranch, and about the same time in the pine forests up in the mountains, at 6,400 feet elevation. He rode a good deal on horseback. He gained some in weight and general health, but his purulent expectoration remained about the same in quantity, and much of the time was mixed with blood. On two different occasions, the expectoration was composed almost entirely of dark clotted blood.

After remaining there four months and seeing no improvement, he became discouraged and returned home by sea, having been absent six months.

He then came again to me to inquire what he had better do. I advised him to submit to thorough local treatment by atomized inhalations, and the employment of rarefied air by means of Waldenburg's pneumatic apparatus*, for the purpose of reducing, by internal traction, the size of the bronchiectic cavity and the emphysematous air cells. This he consented to do, and for four months I gave him the inhalations and employed on him the pneumatic apparatus twice daily.

The inhalations employed consisted mainly of sulpho-carbolate of zinc, extr. krameria, oil of eucalyptus with tar water. At frequent intervals, the use of the above was suspended for one or two days, and argenti nitras (gr. x, aqua dr. 1) was substituted for its stimulating effect. Often when the secretions were tenacious and adhering, the following alkaline solution was of service :

℞

| | | GRAMMA. | |
|---|---|---|---|
| Acidi Salicylici, - | ʒ ss. | 2 | |
| Acidi Boracici, - - | gr. xv. | 1 | |
| Sodii Phosphatis, - | ʒ ii. | 8 | |
| Sodii Bi Carbonatis, - | ʒ ii. | 8 | |
| Acidi Carbolici, - | gr. iv. | | 26 |
| Aquæ Rosæ, - - | ℥ i. | 30 | |
| Aquæ Picis Liquidæ, | ℥ iii. | 90 | |

M.

Compressed air and Sass atomizing tubes were used to nebulize the fluids, which were inhaled through a large and long globular mouth-piece.

After the commencement of this treatment, the expectoration began at once to decrease in quantity, and in two weeks it was

* For a description of this apparatus vide "Ein transportabler pneumatischer Apparat zur mechanischen Behandlung der Respirationskrankheiten. L. Waldenburg. Berl. Klin. Wochenschr. x. 39, 40, 1873." The modifications of this apparatus and the various other apparatuses employed for the same purpose, as those of Berkart, Tobold, Biedert, Störk, Fränkel, Schnitzler, Geigel and Mayr, and others, are fully described by Oertel in his "Handbuch der Respiratorischen Therapie," which is part iv of vol. i. of V. Ziemsen's Handbuch der Allgemeinen Therapie, Leipzig, 1882. Also, vide "Inhalation, its Therapeutics and Practice," by J. Solis Cohen, Phil., p. 43, et seq.

reduced one-half. At the expiration of the time he remained for continuous treatment, it was reduced to six drachms during the twenty-four hours. There was also a corresponding decrease in the size of the cavity, and of the dilated air cells. His general health was greatly improved.

He was now so well and strong that he wished to return to his business, from which he had been absent nearly two years, and I arranged for him to use at his home some apparatus by which he could continue the employment of inhalations and of the rarefied air.

He was, however, still very sensitive and subject to frequent colds, and several mishaps from this cause befell him. The following April, he had lobular pneumonia in the lower lobe of the right lung. Afterwards, a portion of this lobe broke down and was expectorated, leaving a small cavity, which was slowly contracted and healed by the employment of inhalations and pneumatic rarefication.

In May, 1883, he had some pleuritis over this lower right lobe, which yielded to counter-irritation. Since this time, he has been very well and able to attend to his business continuously. He has no dyspnœa on exertion. The emphysema and bronchial dilatations have disappeared or become so small that they cannot be detected. He has, however, still a cough with some mucous expectoration, from which he has not been free since he was two years old. This expectoration is increased somewhat by colds and unfavorable weather, but he keeps it under control by the inhalations, which he has learned to take by himself very thoroughly.

During the present winter, his health is better than it has been during any winter in the past seven or eight years. *

The points of special interest in this case, that have made me think it worthy of being detailed to you, are, the large quantity of pus expectorated daily, the large size of this bronchiectic cavity, and at the same time its obscurity, as shown by the

* (1) Since this paper was read, he has slowly but steadily improved. At the present time, he coughs but very little, and the expectoration is markedly decreased in amount.

different opinions expressed in regard to it, and last, but not least, the positive evidence which this case gives, of the direct value of locally-applied respiratory medicaments, and the marked assistance which mechanical means afford in the reduction of bronchiectic and emphysematous dilatations.

Many other cases of bronchiectasis in a minor degree, associated with emphysema, have come under my observation. The foregoing case will, however, serve as a typical illustration for them all. The treatment adopted was the same or similar to that employed in this case, and gave uniformly excellent results.

Bronchiectasis is always a secondary disease, i. e., it can only be produced when a previously existing disease, either in the interior of the bronchi, or in the surrounding parenchyma of the lung, has brought about a diseased condition favoring or rendering possible the dilatation of a portion or portions of the bronchi, when an inordinate pressure of the column of air in the chest is brought to bear on the weakened parts.

The causes of bronchiectasis may for convenience be divided into—

1. Predisposing.
2. Exciting.

The predisposing may again be divided into—

1. Those affecting the interior of the bronchial tubes, thereby weakening their walls.

2. Those in which the parenchyma of the lung is involved, and which from destruction or shrinkage tend, by the withdrawal of the external support, and by traction on their walls, to dilate or to increase the calibre of the tubes. The principal causes for these conditions are, interstitial pneumonia, the shrinkage of the tissue, resulting from the proliferation of the connective tissue and the cicatricial contraction in the healing of pneumonic excavations, or the traction exerted by pleuritic adhesions.

The exciting cause is the increased air pressure brought to bear on the interior of the tubes, such as that caused by the act of coughing, or by portions of the respiratory tract being occluded or rendered impervious to air, and the air column being undiminished, greater pressure is in consequence brought to bear upon other parts.

Of the predisposing causes that are confined to the interior of the tubes, bronchitis plays the most important role. In fact, it is extremely doubtful if all the other causes combined can be sufficient to cause dilatation of a bronchial tube that has not become weakened by a disease of the tube affecting its walls.

The nutritive changes that take place in the bronchial mucous membrane, when the seat of chronic inflammation, are usually very marked. The two forms of chronic inflammation, which produce conditions directly predisposing to dilatation of the tubes, are the sthenic variety, which produces hypertrophy of the bronchial mucous membrane, and the asthenic form from which thinning of the walls results. The former is sometimes the precursor of the latter, as it often is in mucous tissue of other parts.

The two forms of dilatations of the bronchi most commonly found are the cylindrical or spindle-shaped, and the saccular.

The cylindrical or spindle-shaped variety may be caused by either hypertrophy or atrophy of the walls; while in the saccular forms the walls are almost invariably found to be atrophied.

Hypertrophy of the bronchial mucous membrane may cause dilatation of the tubes by two modes:

By the destruction of the elasticity of muscular fibres of the tubes, the result of the infiltration from the plastic exudate consequent upon the catarrhal inflammation, in which manner the resisting force of the tubes is lessened.

By a decrease in the calibre of the tube, in the hypertrophic form, in proportion as the mucous tissue becomes thickened, which, according to its extent, produces alterations in the equilibrium of the column of air in the chest cavity.

If there is only partial occlusion of the tube, an increased pressure is at once, in proportion to the narrowing of the tube, brought to bear on the unobstructed portion near the obstruction. Reynaud* is of the opinion that the incoming current of air, in these cases, causes the dilatation, while Williams† believes it is mainly the expired current that produces it. In the larger tubes, both causes may be equally effective, as we often find dilatations on both sides of an obstruction ; but in the smaller tubes, the latter cause is unquestionably the most potent. And this accounts for the fact, that, associated with dilatations of the smaller tubes, with narrowing of portions of their calibre, there is almost invariably found emphysematous dilatation of many of the air cells in the immediate neighborhood.

Thus, when a sudden expiratory effort is made, as in the act of coughing, sneezing, blowing, straining at stool, and the like, over-distension of the tubes result in weakening their walls, and dilatation takes place.

In instances in which the tube becomes completely blocked, if the force of the column of air is undiminished, increased pressure is brought to bear on the portion unobstructed, and also on the neighboring tubes, when, if their walls have become weakened by disease and their elasticity impaired, dilatation results. Laennec** believed that lodgment of tenacious mucus in the tube would have the effect to cause cylindrical dilatation of the bronchi.

In some cases, we find both the atrophic and hypertrophic condition present in the same case, which is termed by Biermer†† trabecular degeneration of the walls. In this form, we have alternate elevations and depressions, the ridges being the remaining portions of hypertrophied tissue that have not yet undergone atrophic degeneration.

---

* Mêmoires de l'acadêmie royale de médecine, Tom. IV., Paris, 1835, p. 117, et seq.

† System Pract. Med., Phil., 1841.

** A Treatise on the Diseases of the Chest. Eng. Trans., New York and Phil., 1835, p. 102.

†† Handb. der Spec. Path. u. Therap., 1867, Bd. V., S. 134.

